# The piriform, perirhinal, and entorhinal cortex in seizure generation

**DOI:** 10.3389/fncir.2015.00027

**Published:** 2015-05-29

**Authors:** Marta S. Vismer, Patrick A. Forcelli, Mark D. Skopin, Karen Gale, Mohamad Z. Koubeissi

**Affiliations:** ^1^Department of Neurology, The George Washington UniversityWashington, DC, USA; ^2^Department of Pharmacology, Georgetown UniversityWashington, DC, USA

**Keywords:** piriform cortex, area tempestas, perirhinal cortex, entorhinal cortex, epileptogenesis, temporal lobe epilepsy

## Abstract

Understanding neural network behavior is essential to shed light on epileptogenesis and seizure propagation. The interconnectivity and plasticity of mammalian limbic and neocortical brain regions provide the substrate for the hypersynchrony and hyperexcitability associated with seizure activity. Recurrent unprovoked seizures are the hallmark of epilepsy, and limbic epilepsy is the most common type of medically-intractable focal epilepsy in adolescents and adults that necessitates surgical evaluation. In this review, we describe the role and relationships among the piriform (PIRC), perirhinal (PRC), and entorhinal cortex (ERC) in seizure-generation and epilepsy. The inherent function, anatomy, and histological composition of these cortical regions are discussed. In addition, the neurotransmitters, intrinsic and extrinsic connections, and the interaction of these regions are described. Furthermore, we provide evidence based on clinical research and animal models that suggest that these cortical regions may act as key seizure-trigger zones and, even, epileptogenesis.

## Introduction

The rhinal and piriform cortices are highly epileptogenic regions that are broadly interconnected with other limbic and cortical areas. The functional architecture of these regions involves oscillatory network activity commonly associated with memory processing. As a result, the unique anatomical organization and connections of these regions can also lead to pathological hypersynchronization resulting in seizures. For example, the piriform cortex (PIRC) is part of the primary olfactory cortex, and odor can be a powerful trigger for memory (Neville and Haberly, 2003). The long-range projections of piriform cortex, including interconnections with amygdala, hippocampus, and rhinal cortex place this structure as a crucial hub in the ability of an odor to “trigger a feeling,” stir an autobiographical memory, or trigger seizure activity throughout the limbic system. The rhinal cortex is divided into the entorhinal (ERC) and perirhinal cortices (PRC), which serve as the major cortical communication relays for the hippocampus. Information flow from the rhinal cortex, through the hippocampus, and back to the rhinal cortex is a perfect example of a reverberatory, Hebbian cell assembly. Under normal conditions, this architecture plays a crucial role in the processing of declarative memory. Under pathological conditions, the rhinal-hippocampal loop tends toward excessive propagation and loop-gain amplification, which is a hallmark of temporal lobe epilepsy (Cataldi et al., 2013). Herein, we review: (1) the normal anatomy and functional connectivity of these structures with an emphasis on the features that make them particularly prone to seizure initiation, (2) evidence for epilepsy-related pathology in these sites, and (3) evidence supporting the notion that these features make these sites critical “trigger zones” for seizure initiation in the temporal lobe.

## Piriform cortex (PIRC)

### PIRC functions

The PIRC is a part of olfactory cortex and its typical activities are olfactory processing and memory coding (Neville and Haberly, 2003). The posterior PIRC is activated in tasks that require categorization of odors (Howard et al., 2009). Closely associated with the PIRC is the endopiriform nucleus (EPN), a large mass of multipolar cells located within the PIRC and has been implicated in seizure generation and propagation (Piredda and Gale, 1985, 1986; Demir et al., 1998, 1999, 2001; Behan and Haberly, 1999; Mraovitch, 2003; Laufs et al., 2011; Vaughan and Jackson, 2014). Although its functions remain largely unknown, the EPN may have a role in integrating gustatory and olfactory sensory information (Sugai et al., 2012). Furthermore, PIRC and EPN may also produce olfactory and gustatory semiology when affected by ictal discharges.

### Pirc anatomy and histology

The PIRC is a large, cytoarchitectonically homogeneous area that extends over the ventrolateral surface of the forebrain, and constitutes a major component of primary olfactory cortex (Neville and Haberly, 2003). The PIRC consists of three layers: superficial plexiform layer (layer I), cell body layer (layer II), and layer III containing soma and fibers (Sperber et al., 1998). Adjacent to layer III is the well-defined EPN comprised of multipolar cells (Tseng and Haberly, 1989). The EPN is also referred as layer IV of the PIRC since it spans the entire rostro-caudal dimension of the PIRC. The dorsal region of this nucleus consists of densely packed cells while the ventral region has more diffusely arranged cells (Krettek and Price, 1977). The ventro-rostral aspect of the PIRC encompasses a functionally defined and histologically distinct chemoconvulsant trigger zone, located deep within the anterior PIRC, named area tempestas (AT) (Piredda and Gale, 1985, 1986; Ekstrand et al., 2001). Compared to the rest of the PIRC, studies have demonstrated that AT possesses a decreased number of GAT-1 immunoreactivity with decreased density of GABAergic inputs at the axonal initial segments along with decreased concentration of cholecystokinin positive interneurons (Ekstrand et al., 2001). EPN shares a number of properties with the PIRC such as excitatory discharges, hyperexcitability, and high speed of signal propagation. Together these features lead to a high potential for epileptogenecity (Hoffman and Haberly, 1991, 1996; Demir et al., 1998, 2001). Moreover, both the PIRC and EPN are exceedingly vulnerable to excitotoxic injury, and represent one of the earliest sites of neuronal loss after status epileptics (Scholl et al., 2013). As discussed below, the extensive interconnections of the PIRC/EPN with other highly excitable brain regions may allow for the generation and spread of epileptiform activity within PIRC/EPN and throughout the brain (Löscher and Ebert, 1996).

### PIRC connections

**Figure 1:**
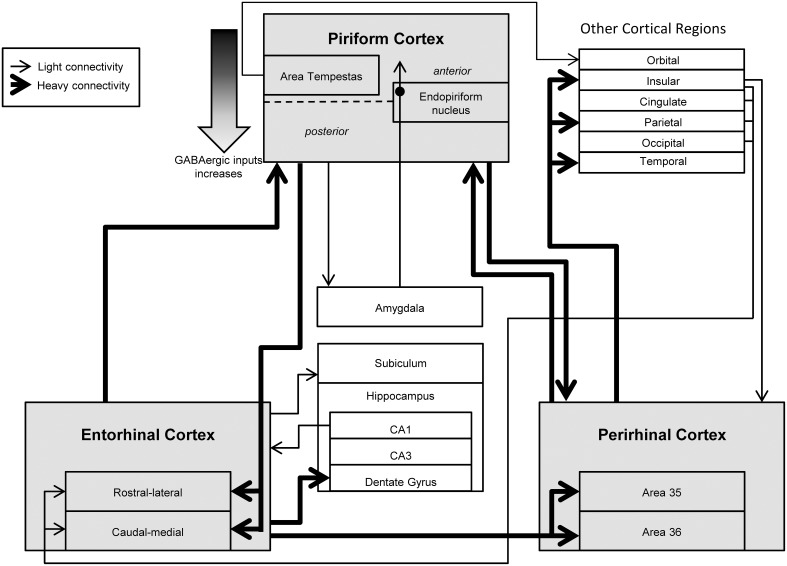
**Overview of the functional connectivity of piriform, perirhinal, and entorhinal cortices**. Many studies have investigated the connectivity of the piriform (PIRC), perirhinal (PRC), and entorhinal cortices (ERC) along with their relationship with other cortical and limbic structures. These regions are highly interconnected with each other and mediate bidirectional information from higher processing regions. Abnormal activity from these regions is hypothesized to cause hyperexcitability and excessive propagation associated with temporal lobe epilepsy along with perceptual disturbances such as hallucinations and auras.

The integrative connections between PIRC and several limbic nuclei likely facilitate propagation of ictal activity between these structures (Figure 1). Afferents from the ERC constitute the most dense connections of PIRC (Burwell and Amaral, 1998). In turn, the PIRC and EPN send efferent projections to the ERC (Luskin and Price, 1983a; Agster and Burwell, 2009). There is also a direct connection from the PIRC to the amygdala (Veening, 1978; Wakefield, 1980) and a diffuse polysynaptic connection from the amygdala to the PIRC via the EPN (Krettek and Price, 1977), which may have significant implications for temporal lobe epilepsy (TLE). There are also pathways from the PIRC to the subiculum (Krettek and Price, 1977; Luskin and Price, 1983a) and to areas 35 and 36 of PRC (Agster and Burwell, 2009).

There are significant differences in the connectivity of the anterior and posterior subregions of the PIRC (Haberly, 2001; Schwabe et al., 2004), suggesting functional specialization within the PIRC (Illig, 2005), which may be of particular importance to the functional topography related to seizure initiation. For example, local circuit GABAergic transmission appears to be stronger in the posterior sub-region of the PIRC and become increasingly less prominent more anteriorly (Löscher and Ebert, 1996; Haberly, 2001), Interestingly, AT, crucial to seizure generation (Piredda and Gale, 1985), is located in the anterior aspect of the PIRC, and has low density of GABAergic inputs to the axon initial segments (Ekstrand et al., 2001). Unlike the rest of the PIRC that receives olfactory input from mitral cells only, the AT also receives direct projections from tufted cells in the olfactory bulb (Ekstrand et al., 2001) and provides most input from the PIRC to ventrolateral orbital cortex. These connections are both directly and via di- and trisynaptic connections incorporating pre-endopiriform nucleus and submedial and mediodorsal thalamus (Ekstrand et al., 2001; Illig, 2005). This interconnectivity may be important for seizure propagation and generalization.

Projections from the PIRC and EPN are very diffuse. Single neurons within posterior PIRC send axonal arbors to the insula, PRC, ERC, amygdala, and olfactory tubercule, with a tendency to have no overlap with the arborization patches created by neighboring neurons (Johnson et al., 2000). Projections of the EPN parallel those of the PIRC, targeting ERC, PRC, insular, orbital, and other neocortical structures (Behan and Haberly, 1999). The EPN is the major route for information transfer along the rostrocaudal axis of PIRC/EPN and may facilitate seizure propagation longitudinally within this structure (Behan and Haberly, 1999). Moreover, EPN may play an especially important role in the spread of activity from EPN and PIRC to other cortical regions, because the EPN has a more diffuse topographic and laminar organization of axon terminals than does PIRC (Luskin and Price, 1983a,b; Behan and Haberly, 1999). The extensive interconnectivity of EPN and PIRC with other epileptogenic sites within the temporal lobe (e.g., ERC, PRC) may provide a loop for positive feedback of activity and subsequent generalization of epileptic discharges.

## Perirhinal cortex (PRC)

### PRC functions

The PRC is involved in complex memory functions (Rosen et al., 1992; Zola-Morgan et al., 1994; Wiig et al., 1996), such as object recognition, sensory representation, and spatial orientation (Murray and Richmond, 2001). Damage to PRC results in deficits in complex visual discrimination (Ryan et al., 2012), object recognition memory (Meunier et al., 1993; Zola-Morgan et al., 1994), and attention to visual stimuli (Bucci and Burwell, 2004). The PRC represents a component of the ventral visual stream, with interconnections to secondary visual regions being more robust than those with primary visual cortex (Agster and Burwell, 2009). This may support its involvement in higher visual information processing and memory (Barbeau et al., 2005) and may account for complex visual hallucinations in some temporal lobe seizures. Induction of theta activity in the PRC network results in the recall of vivid memories (Barbeau et al., 2005) and therefore, can represent a substrate for visual auras that accompany some TLE. PRC damage may also account for the feeling of *déjà vu* reported by a subset of TLE patients during the aura of their seizures (Martin et al., 2012). Extensive damage to PRC in epilepsy may explain the cognitive decline, including primarily memory deficits (Shatskikh et al., 2006) that occur in some patients with chronic TLE.

### PRC anatomy and histology

The human PRC is located along the collateral sulcus in the ventromedial portion of the temporal lobe. Laterally, it borders the ERC with which it comprises the parahippocampal gyrus (Insausti et al., 1987). On the basis of cytoarchitectural, immunohistochemical, and physiological criteria, the PRC is further subdivided into two parallel cortical bands, areas 35 medially, and 36 laterally (Insausti et al., 1987; De Curtis and Paré, 2004; Saleem et al., 2007).

### PRC connections

#### Afferents

Both Areas 35 and 36 of PRC receive substantial input from ERC (Figure 1). In addition to this input, PRC receives projections from ventral temporal association areas, PIRC, and other limbic-cortical areas (e.g., insula). The relative abundance and distribution of these inputs varies as a function of subregions of PRC (i.e., Area 35 vs. 36) (Burwell and Amaral, 1998).

#### Efferents

The PRC projects back to many of the same regions it receives input (Luskin and Price, 1983a,b; Burwell and Amaral, 1998; Agster and Burwell, 2009). For example, the PIRC, insula, and subcortical limbic structures all have reciprocal connections with the PRC. Just as the pattern of afferents differs between Areas 35 and 36, the patterns of efferents also differ (Agster and Burwell, 2009). The PRC may represent a route by which seizure activity can propagate to distal cortex due to the heavy connections with the insula and sensory domains within frontal, temporal and parietal cortices. These projections may also help explain sensory symptoms associated with TLE including gustatory or autonomic symptoms related to insular cortex activation. There is a high degree of specialization within various subregions of the PRC (e.g., rostral PRC projects heavily to somatosensory cortex, while caudal PRC projects heavily to visual cortex) (Agster and Burwell, 2009). A relatively underexplored question is the degree to which topographic activation of the PRC may be associated with different patterns of propagation and/or symptoms associated with TLE.

The PRC is a “gate-keeping” area that mediates bidirectional information transfer between temporal neocortex and ERC (Pelletier et al., 2004). It is worth noting that while most communication takes place through a slow, stepwise progression through cortical areas, a minor portion of axons bypass the PRC (De Curtis and Paré, 2004) directly activating the lateral ERC. Moreover, some ERC axons extend to area 35 and the temporal neocortex. These projections may allow for spread of pathological excitatory activity by circumventing the PRC gate. In conditions such as chronic epilepsy, the reciprocal interconnectivity of PRC with other hyperexcitable structures positions the PRC as a relay region within the limbic network for the generation and propagation of epileptiform activity.

## Entorhinal cortex (ERC)

### ERC functions

The ERC, located in the anterior parahippocampal gyrus, serves as the major interface between the hippocampus and sensory cortices (Insausti et al., 1987). Its close connection with cortical regions involved in the processing of visual and spatial information suggests that the ERC is an important substrate for visuo-spatial functioning (Burwell and Amaral, 1998; Killian et al., 2012). Moreover, hippocampal memory function depends on an intact ERC (Lu et al., 2013) and appears to be well-conserved across species. For example, human neuroimaging studies have found correlations between parahippocampal activity and spatial working memory (Courtney et al., 1996), and with learning and recall of topographic information (Aguirre et al., 1996). In monkeys, single neurons within the ERC fire in response to visual input from specific locations in the environment, forming memories of spatial environments (Rolls and O'Mara, 1995). Moreover, “grid neurons” of the ERC have been well-described in rodents and primates (Sargolini et al., 2006; Killian et al., 2012; Navratilova et al., 2012; Lu et al., 2013). As with PIRC and PRC, the extensive connections between ERC and other limbic brain regions suggest it is a major relay for seizure activity.

### ERC anatomy and histology

In humans, the ERC, consisting of Brodmann areas 28 and 34, is situated bilaterally between the sulcus semiannularis and sulcus rhinalis in the rostral parahippocampal gyrus (Leichnetz and Astruc, 1976; Amaral et al., 1987). It is an allocortical structure, consisting of six layers, with a striking paucity of cells in layer IV (lamina dessicans), which separates the outer cell-rich layers (V and VI) from the inner cell-rich layers (II, and III) (Amaral et al., 1987; Insausti et al., 1995). Layer III may be particularly important for seizure propagation as it consists of pyramidal projection neurons that target the hippocampus and other regions. For a detailed review of ERC anatomy and cytoarchitectonics, see Insausti (1993).

Like the PRC, the ERC is divided in two major sub-fields, the rostral-lateral (LERC) and the caudal-medial ERC (MERC) (Dolorfo and Amaral, 1998; Uva et al., 2004). These two divisions differ in their histology, morphology and physiology. The LERC has a number of morphological inhomogeneities and the neurons are organized in patches surrounded by thick radially-oriented bundles of fibers. Conversely, the MERC is more reminiscent of the neocortex, with a distinctly laminar appearance and columnar organization of neurons and fibers (Amaral et al., 1987). As will be discussed in the later sections, this laminar structure may have a special role in signal amplification and epileptogenesis.

### ERC connections

#### Afferents

A major input to both LERC and MERC originates within PIRC and accounts for about one-third of the input to these regions (Figure 1). Other inputs originates in temporal and frontal cortices. Less abundant sources of input include insular, cingulate, parietal, and occipital cortex, with the relative abundance of these inputs varying between the MERC and LERC.

#### Efferents

In addition to its rostro-caudal organization, the ERC is also organized into bands that project to different septo-temporal levels of the dentate gyrus (Dolorfo and Amaral, 1998; Agster and Burwell, 2009). As other structures discussed in this review, the distribution of efferent projections differs between subregions of ERC. PIRC is the major recipient of input from both subdivisions of ERC of projections to inferotemporal, retrosplenial, posterior parietal, frontal, and insular cortices originating in medial and lateral domains. Similar to PIRC and PRC, many of the connections from ERC are reciprocated (Biella et al., 2002a,b). This ability for bidirectional information transfer, while presumably important for normal information processing, is yet another example of a cortico-cortical loop that can amplify and propagate seizure discharges.

The hippocampus is a key area to seizure propagation and has a series of parallel ERC-hippocampus-ERC loops that underlie memory functions (Andersen et al., 1971; Acsády and Káli, 2007; Kibler and Durand, 2011). In the TLE, one of the most common types of medically intractable epilepsy (Engel et al., 2003, 2012), ictal synchronization within hippocampus is a pre-requisite to spread of activity to other cortical areas (Bartolomei et al., 2001; Pallud et al., 2008; Umeoka et al., 2012) and the hippcampal-ERC communication may serve as a critical substrate for propagation of epileptic activity of ERC origin to hippocampus.

### Intrinsic circuitry

Despite histological data that strongly suggest the presence of substantial fiber tracts between the ERC and PRC, the functional strength of connections in the healthy brain is debatable (Biella et al., 2002a). Previously, the PRC was considered to be a relay nucleus for the bidirectional communication between the neocortex and the ERC. However, more recent data suggests the PRC is an important center for feed-forward inhibition of the ERC (Finch et al., 1988; Biella et al., 2002a; De Curtis and Paré, 2004; Pelletier et al., 2004). Extracellular recordings and optical imaging studies demonstrated that PRC–ERC and ERC–PRC interactions produce only limited neuronal activation with a relatively low probability for the transfer of neocortical inputs to ERC. This highly restricted and controlled information transfer is essential in a recurrent circuit to prevent loop-gain amplification. Therefore, in the healthy brain, the excitatory activity of the ERC is controlled by inhibitory interactions in the network that are activated by extrinsic projections. The PRC may act as a “gate” for spread of excitatory activity between the ERC and neocortex. While conditions of normal hyperexcitability can facilitate the propagation of olfactory-driven activity from the ERC to the PRC (Federico and MacVicar, 1996; Kelly and McIntyre, 1996), malfunction of intrinsic control mechanisms can result in hyperexcitability of the ERC and, consequently, epileptic seizures.

## Summary of functional anatomy

Together, PIRC/EPN, PRC, and ERC form a unique and widely connected network that is ideally organized to engage the hippocampus (and the rest of the limbic system) in rhythmic activity. While this rhythmic activity is a part of normal brain function, malfunctions in the circuitry can easily lead to pathological synchronization and seizure spread. Seizures do not propagate randomly through the brain, but rather utilize circuits that normally support highly controlled recurrent activity. Through this network, activity can spread to distal cortical and subcortical regions along pathways that are also used in normal motor system (Haberly and Bower, 1989; Wilson and Stevenson, 2003; McIntyre and Gilby, 2006; Howard et al., 2009).

In the sections above, we have described the anatomical features that position PIRC/EPN, PRC and ERC as sites through which seizure discharges can amplify and propagate. While these regions are capable of autonomous generation of ictal discharges, the importance of their interconnections is underscored by data demonstrating a significant reduction in ictal activity when the connections between these regions are disrupted (De Guzman et al., 2004). These observations imply that the communication between the ictogenic structures is a key component for amplification and synchronization of ictal bursts and may have particular relevance to understanding the effects of temporal lobe resection for the treatment of epilepsy. Even “selective” amygdalohippocampectomy (aside from radiofrequency lesions) involve resection of the anterior aspects of the middle and inferior temporal gyrus, disrupting many connections intrinsic to the temporal lobe in addition to removing temporal cortical tissue, amygdala, and hippocampus.

Below we discuss conditions that may lead to failures in the intrinsic gating properties of these structures. These pathologies and subsequent aberrant gating are hypothesized to underlie the emergence of spontaneous seizures in chronic epilepsy.

## Morphological and histological changes due to epilepsy

The transition from normal network activity to hypersynchronous activity characteristic of a seizure may indicate a subtle change in the interconnections or functions within PIRC, PRC, ERC, or the broader limbic network. A common histopathological finding in TLE is neuronal loss and inflammation. It is possible to speculate that these changes may result in the network perturbations necessary for epilepsy or the comorbid memory and cognitive deficits. However, the degree to which these histopathological findings are a cause or an effect of recurrent seizures remains a matter of debate. In the section below, we will discuss evidence for neuropathology in PIRC, PRC, and ERC in the context of epilepsy and epileptogenic insults.

### Neuroimaging

In patients with TLE, volumetric analyses reveal volume loss of the anterior temporal lobe on the epileptic side (Lencz et al., 1992; Jutila et al., 2001; Bonilha et al., 2003; Martin et al., 2012), bilateral reduction in temporal neocortical gray matter, and reduction in temporal white matter (Lee et al., 1998). Changes in volume of temporal cortex appear to be specific to TLE, and are not seen in extratemporal epilepsy (Jutila et al., 2001; Bonilha et al., 2003). Analysis of substructures within the TLE has also reveal volumetric changes, including in the hippocampus, PRC, and ERC (Bonilha et al., 2003). In one study, 52% of patients with medically intractable TLE have reduction in ipsilateral ERC volume (Jutila et al., 2001). Moreover, the extent of atrophy in ERC correlates with ipsilateral hippocampal volume and the duration of epilepsy (Jutila et al., 2001).

While it remains controversial if damage to piriform and rhinal structures leads to epilepsy, or rather if the recurrent seizures characteristic of epilepsy lead to damage, some patients have atrophic tissue in the temporal lobe at what appears to be the site of seizure origin. For example, in some patients with TLE, ERC appears to display epileptiform activity before the hippocampus, but only in patients with atrophy of ERC (Bartolomei et al., 2005). Interestingly, the degree of atrophy in ERC correlates with hypersynchronization between ERC and hippocampus. These data suggest that the atrophic ERC may be the epicenter of epileptogenic activity with dissemination to other limbic structures via an elaborate network of neuronal connections of the ERC.

### Histopathology

Morphological changes, such as neuronal loss and reactive gliosis, are prominent both in hippocampus and extrahippocampal areas (e.g., ERC, PRC) in patients with TLE (Du et al., 1993; Jung et al., 2009; Scholl et al., 2013). Animal models parallel these findings with ERC, PIRC, and PRC showing some of the most severe damage as soon as 72 h after status epilepticus (Scholl et al., 2013). Even sublayers of these structures show differential vulnerability. For example, there is a profound and relatively selective loss of neurons within layer III of the ERC in patients with TLE (Du et al., 1993). In animal models, loss of neurons within layer III of ERC can occur as soon as 24 h after injury, and progresses with time (Drexel et al., 2012). Morphological changes in the CA1, CA3, and the hilus of the dentate gyrus showed a continuous degeneration of neurons up to 7 days following experimental status epilepticus (Langer et al., 2011). Synaptic reorganization and atrophy of layer III of the ERC produces a robust change in evoked and spontaneous activity in parts of the ERC and hippocampus that result in increased excitability and susceptibility to reentrant activation of the hippocampal–ERC loop via disinhibition of local networks in CA1 (Empson and Heinemann, 1995).

Interestingly, neuronal loss in layer III shows a marked sparing of parvalbumin-expressing interneurons within the medial ERC (Du et al., 1995) with predominate loss of glutamatergic neurons (Drexel et al., 2012) suggesting that a simple depletion of inhibition is insufficient to account for the hyperexcitable phenotype. However, others have described depletions of GABAergic neurons and a reduction in the number of GABAergic synapses after status epilepticus (Kumar and Buckmaster, 2006) suggesting that loss of inhibitory tone may contribute to a hyperexcitable state permissive to seizures. In addition, low-threshold-spiking (LTS) and fast-spiking (FS) interneurons within the medial entorhinal cortex creates a recurrent inhibitory network which is central to stability in grid formation (Couey et al., 2013).

In addition to the cell loss associated with excitotoxic seizure injury (i.e., status epilepticus), the activation and proliferation of microglia may contribute to secondary injury. For example, within the rodent hippocampus, prolonged seizure activity can trigger the proliferation and differentiation of neural progenitor cells (Parent et al., 1997, 2002), resulting in the aberrant and ectopic neurogenesis in the hilus of the hippocampus. However, in extrahippocampal sites (i.e., PIRC, ERC) cellular proliferation and differentiation manifests as increased numbers of astrocytes and oligodendrocytes (Jung et al., 2009). The production of chemokines by prolonged seizures may be of particular importance for triggering aberrant neurogenesis in the hippocampus, reactive gliosis, and astrocytosis in the temporal cortex (Jung et al., 2009).

As soon as 5 h after generalized seizures, a marked elevation in the expression of IL-1β can be detected in the endothelial cells of the blood vessels in the PIRC (Mraovitch, 2003). IL-1β is a modulator of inflammatory response and apoptosis (Darlington et al., 1986; Hartung et al., 1989). The effect of IL-1β and TNF-α on hyperexcitability and seizure development have been shown in several studies (Vezzani et al., 2011). However, some evidence suggest that TNF-α may decrease glutamate-mediated excitotoxicity via NF-kappaB-dependent up regulation of K2.2 channels (Dolga et al., 2008). In the PRC, astrocytes and microglia also express IL-1β and display vascular endothelial expression of TNF-α, another vital component of inflammatory response (Darlington et al., 1986). Within 6 weeks after generalized seizures, histopathological changes such as reactive astrocytosis and irregular microvascular proliferation appear in the allocortex including the PIRC, PRC, and ERC. Perivascular infiltration of C3 positive leukocytes has also been observed (Jamali et al., 2006). These changes are suggestive of inflammation and remodeling and provide a link between the epilepsy-induced inflammatory vascular processes, gliosis, and subsequent allocortical atrophy (Mraovitch, 2003).

Reactive astrocytes and microglia are other markers of inflammatory process and appear in the areas of neuronal deterioration as early as 24 h after the induction of kainate-induced animal model of status epilepticus. Inflammatory cells continue to rise, becoming prominent after 8 days with their number peaking at 30 days and subsequently declining almost to the baseline by 3 month after status; the initial rise in these cells mirrors the time course of spontaneous seizures in this model (Drexel et al., 2012).

Markers of neuronal remodeling and synaptic plasticity, such as polysialylated neural cell adhesion molecule (PSA-NCAM), are increased in ERC and hippocampus from human patients with TLE (Mikkonen et al., 1998) and in rodent models of TLE (Pekcec et al., 2008; Rossi et al., 2013). Changes in neuromodulatory systems and co-transmitters also occur in TLE with striking increases in NPY and somatostatin immunoreactivity and dysregulation of NPY receptor expression in PIRC and ERC (Kato et al., 1983; Vezzani et al., 1996; Kang et al., 2000; Jamali et al., 2006). In TLE and its models, it is difficult to determine if changes in plasticity represents a compensatory or decompensatory response to initial injury. For a more detailed review of the role of inflammation in TLE, see Vezzani et al. (2011).

In summary, epileptic seizures are associated with significant alterations in the histology of the PRC, PIRC, and ERC. Cell loss, inflammation, and altered gene expression may shift the balance between inhibition and excitation to create a pro-epileptic environment. Because these structures gate information transfer within the temporal lobe and extratemporal targets, atrophy in these regions may facilitate seizure propagation throughout the forebrain.

## Epileptogenecity

### Piriform cortex—area tempestas

The deep anterior piriform cortex contains a highly circumscribed and functionally-defined chemoconvulsant trigger zone called area tempestas (AT). From this site, unilateral microinjection of picomole amounts of GABA receptor antagonists or glutamate receptor agonists can trigger limbic motor seizures in rats and non-human primates (Piredda and Gale, 1985, 1986). More recently, neuroimaging approaches have found focal fMRI activation and reduced GABA_A_ receptor binding in the anterior piriform cortex of human patients with focal epilepsy (Laufs et al., 2011). Not only is activation of AT sufficient to trigger complex partial seizures in animals, it also appears to be necessary for the response to systemically applied chemoconvulsants (i.e., bicuculline) (Piredda and Gale, 1985, 1986) and nerve agents (i.e., soman) (Myhrer et al., 2006, 2008). Enhancement of GABA-mediated inhibition within this structure markedly attenuates seizure activity evoked by systemic chemoconvulsant administration (Piredda and Gale, 1985, 1986). In adult animals, AT is generally accepted as a zone of high epileptogenicity, while in immature rats, there was no difference between the rate of kindling from AT and PIRC (Sperber et al., 1998). These findings suggest that developmental changes in excitability within these subregions may modulate their epileptogenicity.

Some studies suggest a crucial role for the deep layers of the PIRC and/or the EPN in the induction of epileptiform bursting in slice preparations (Hoffman and Haberly, 1991, 1993; Doherty et al., 2000). While *in vitro* studies have demonstrated that PIRC/EPN slices are highly excitable across the entire anteroposterior axis, *in vivo* studies demonstrate that the anterior PIRC/EPN has the highest susceptibility to seizure initiation (Piredda and Gale, 1985). The differences between these findings may be explained by the nature of the slice model utilized in which the entire slice was disinhibited by reduction in extracellular chloride (Demir et al., 1999).

Seizure propagation from AT requires both the posterior PIRC and PRC; pharmacological inhibition of these structures significantly attenuates the severity of seizures evoked from AT (Halonen et al., 1994). Likewise, blockade of excitatory neurotransmission within PRC suppressed amygdala kindled seizures (Holmes et al., 1992). Together, convergent evidence from experiments in acute slices, pharmacological manipulation of the rat brain *in vivo*, and human neuroimaging provide evidence that PIRC, and in particular, AT, is a crucial site for seizure initiation, and PRC is a key relay for seizure propagation within the limbic network. In the sections below we will outline additional evidence from pharmacological and kindling studies supporting these conclusions.

### Kindling

Animal epilepsy model studies using kindling suggest that PIRC and PRC have particularly low thresholds for epileptogenesis; kindling from these structures occurs at a faster rate (i.e., with fewer stimulations necessary to trigger Stage 5 seizures) as compared to other forebrain structures (McIntyre and Plant, 1993; McIntyre et al., 1999). Interestingly, the faster rate of kindling from PIRC/PRC was seen in both kindling-prone and kindling-resistant rat strains (McIntyre et al., 1999). Within PIRC, cells in Layer III have the lowest afterdischarge threshold, but electrode placement within the EPN results in the fastest rate of kindling (Löscher and Ebert, 1996). PIRC lesions have only mild effects on kindling: Lesions to the anterior PIRC have no effects, lesions to the posterior PIRC increase afterdischarge thresholds but do not alter kindling rate, and lesions to central PIRC increase the number of Stage 3 seizures before animals fully kindled (Wahnschaffe et al., 1993; Schwabe et al., 2000). Low frequency stimulation of the central PIRC has a similar effect of hindering generalization of focal seizures (Yang et al., 2006) and decreasing the duration and stage of seizures in fully kindled animals. Thus, the PIRC is readily capable of kindling and plays an important role in development and generalization of seizures of different origins, which may have tremendous therapeutic potential. The PIRC may be the site of interictal discharge origination within the limbic circuitry of kindled animals—regardless of the site of kindling (Kairiss et al., 1984). Within PRC, kindling not only occurs with the fewest stimulations of any site within the forebrain, but the latency from stimulation to behavioral seizures is very brief. This brief latency, along with extensive projections from PRC to frontal cortex led McIntyre and Kelly to propose that PRC is a crucial relay that clonic motor convulsions are triggered after limbic seizures (Kelly and McIntyre, 1996). Supporting this hypothesis, damage to perirhinal cortex prevents motor seizures evoked by hippocampal kindling (Kelly and McIntyre, 1996). While both the PIRC and PRC are highly susceptible to generating epileptiform discharges, the PRC is generally involved in generating seizures in naïve animals, though the role of epilepsy trigger zone shifts to PIRC as a result of the process of epileptogenesis (McIntyre and Plant, 1993).

Epileptogenesis relies heavily on synaptic plasticity. For example, tetanic stimulation of the white matter of the PRC and Schaffer collaterals of the hippocampus suggesting reciprocal long-term potentiation between the hyperexcitable structures pertinent in epilepsy (Supcun et al., 2012). Short and long-term plasticity also implicate changes at the receptor level and refer to the alteration in both number and composition of certain receptor groups leading to decreased inhibition of some brain structures and increasing their propensity to produce excitatory bursts. An increase in the density of α-1 adrenergic receptors (Gundlach et al., 1995), modifications in the composition of NMDA and AMPA glutamate receptors (Supcun et al., 2012), and changes in morphology and number of gluzinergic (zinc-containing glutaminergic) terminals in PRC and PIRC (Galvis-Alonso et al., 2004) occur as part of the changes of synaptic plasticity elicited by induction of long term potentiation. These changes are believed to play a role in epilepsy by facilitation of further seizures. Garcia-Cairasco and colleagues used acoustic kindling to observe long-term morphological changes (Garcia-Cairasco et al., 1996). In the groups that underwent long-term kindling and 60 days of recovery, staining revealed mossy fiber sprouting in the ventral hippocampus and a significant change in the optical density of PRC in susceptible animals as compared to resistant ones suggesting that permanent structural changes are present soon after the initial seizure. Changes on the synaptic and cellular level collectively create an imbalance between excitation and inhibition in PRC and PIRC which are predisposed to excitation and have the infrastructure for synchronization of neuronal activity that amplifies and propagates excitatory signals throughout the neocortex resulting in TLE with its other comorbidities.

Hippocampal kindling also induces long-term changes in the dynamics within the amygdala-piriform-perirhinal slice. After hippocampal kindling, spontaneous discharges in the slice originated within PIRC, whereas in slices from control animals, discharges originated within PRC. Unlike amygdala kindling, which produced strictly unilateral changes in excitability, kindling of dorsal hippocampus generated bilateral alterations. Even partial hippocampal kindling was linked to presence of spontaneous spikes in the PRC under hyperexcitable conditions (McIntyre et al., 2000). These findings show a change in the region of seizure onset are consistent with *in vivo* observations that early in epilepsy seizures began with focal onset, while later seizures had more diffuse onset (including hippocampus, amygdala, piriform and entorhinal cortices) (Bertram, 1997). This provides evidence for the hypothesis that epileptogenesis is an active process, initiated in hyperexcitable trigger zones that progressively involves more structures with wider network distribution over time and emphasis should be placed on early therapeutic intervention. As discussed in the section on histopathology, this process is accompanied by structural and functional changes in the seizure network, including loss of functional complexity in the interneurons of epilepsy trigger zones (Gavrilovici et al., 2012).

## PIRC and PRC pharmacological studies

### NMDA receptor

The role of N-methyl-D-aspartate (NMDA) receptors in the generation and propagation of seizures differs between anterior PIRC (i.e., AT), posterior PIRC, and the PRC. Within the AT, blockade of NMDA receptors prevents bicuculline-evoked seizures (Halonen et al., 1994; Fornai et al., 2005) and attenuates amygdala-kindled seizures (Holmes et al., 1992). Similarly, within the PRC, NMDA receptor activation is necessary for seizure propagation. In contrast, blockade of NMDA receptors in posterior PIRC was without effect on AT-evoked seizure propagation (Piredda and Gale, 1986; Halonen et al., 1994; Tortorella et al., 1997). The pattern differs slightly in the non-human primate: blockade of NMDA receptors within AT using 2,3-dihydroxy-6-nitro-7-sulfamoyl-benzo (f)-quinoxaline (NBQX) or 3-(2-carboxypiperazin-4-yl)propyl-1-phosphonic acid (CPP) does not significantly protect against AT-evoked complex partial seizures (Malkova et al., 2015). Seizures evoked by soman, a nerve agent inhibitor of acetylcholinesterase, show some resemblance with AT-evoked seizures in terms of the profile of NMDA receptor involvement across regions. For example, NMDA receptor blockade in the posterior PIRC offered no protection against soman-induced seizures, while NMDA receptor blockade in the PRC more than doubled the latency to seizure onset after soman exposure (Myhrer et al., 2010). This pattern differs with respect to NMDA receptor antagonism in AT, which is without effect against soman administration (Myhrer et al., 2008). In the *ex vivo* guinea pig brain, seizure activity evoked with the potassium channel blocker 4-aminopyridine (4-AP) is actually worsened by blockade of NMDA receptors. Finally, in slice models, blockade of NMDA-receptor mediated neurotransmission attenuates ictal and interictal-like activity evoked by 4-AP (Salah and Perkins, 2011), by high extracellular potassium concentrations (Traynelis and Dingledine, 1988), and spontaneous and evoked discharges from pilocarpine (Benini et al., 2011). Thus, NMDA mediated neurotransmission plays a pivotal role in the propagation of seizure activity within the PIRC/PRC network.

### AMPA

Alpha-amino-3-hydroxy-5-methylisoxazole-4-propionic acid (AMPA) receptor mediated neurotransmission is critical for seizure initiation and propagation within anterior and posterior PIRC along with PRC. For example, systemic administration of the AMPA receptor antagonist, NBQX, produced a dose-dependent decrease in the severity of seizures evoked from AT (Tortorella et al., 1997). Moreover, microinjection of NBQX into either AT, posterior PIRC or PRC antagonized AT-evoked seizures with doses as low as 500 pmol. Unlike the role of NMDA receptors, the role of AMPA receptors in this circuit is conserved between rats and monkeys: AMPA receptor blockade within AT suppressed AT-evoked seizures in the monkey (Malkova et al., 2015). Blockade of AMPA receptors within PRC is also effective against soman-evoked seizures (Myhrer et al., 2010). In the guinea pig model, blockade of AMPA receptors significantly attenuated seizures triggered by 4-AP within piriform cortex, entorhinal cortex, and hippocampus (Carriero et al., 2010).

AMPA receptor desensitization within AT appears to play a pivotal role in controlling the duration of seizure activity. For example, co-application of cyclothiazide (which blocks AMPA receptor desensitization) and bicuculline triggers a self-sustaining status epilepticus (Fornai et al., 2005). Moreover, overexpression of slow desensitizing (GluR1 flip), but not fast desensitizing (GluR1 flop) AMPA receptors within AT significantly increased the severity of bicuculline evoked seizures. In all cases, AT-evoked status epilepticus was insensitive to NMDA receptor blockade but was prevented by AMPA blockade (Fornai et al., 2005). Within the monkey, given the relative propensity of AT-evoked seizures to result in status epilepticus, and the insensitivity of AT-evoked seizures to NMDA receptor activation, it could be speculated that AMPA receptors within PIRC may be slow to desensitize.

At the level of synaptic physiology, AMPA antagonism with DNQX can eliminate epileptiform events in the slice (Hoffman and Haberly, 1996). This effect is associated with decreased buildup in multiunit activity preceding epileptiform discharges. Interestingly, this effect occurs at a concentration below that required to completely block monosynaptic EPSPs suggesting that the disruption in epileptiform activity by AMPA blockade results from disruption of a multisynaptic process. EPSPs mediated by AMPA receptors may be the basis for the epileptiform events in the PIRC. In contrast to NMDA receptors, which contribute but are not indispensable for seizure generation, AMPA receptors appear to be a key component to triggering seizures.

Together, the experimental data discussed above indicate that AMPA-mediated (as compared to NMDA-mediated) neurotransmission plays a relatively larger role in seizure generation within the PIRC/PRC network.

### GABA

GABA-mediated (in particular GABA_A_) neurotransmission plays a critical role in controlling hyperexcitability of neuronal circuits. Disinhibition of hyperexcitable regions by loss of GABAergic inhibition has been long proposed as a mechanism of seizure propagation (Prince and Wilder, 1967). As previously discussed, epileptogenic insults can change the balance between excitation and inhibition within PIRC/PRC in several ways, including by loss of inhibitory neurons. Multiple studies have demonstrated the pivotal role for GABA_A_-mediated neurotransmission in PIRC and PRC for seizure control. For example, picomole amounts of GABA antagonist administered within AT or in posterior PIRC can trigger limbic motor seizure in rat (Piredda and Gale, 1985). In the *ex vivo* guinea pig brain, microinjection of bicuculline in the anterior PIRC results in interictal spiking and spontaneous epileptiform potentials (De Curtis et al., 1994). Interestingly, in a PIRC slice model of epileptiform activity evoked by 4-AP blockade of GABA_A_ mediated transmission abolishes ictal-like discharges, but leaves periodic interictal activity intact (Panuccio et al., 2012).

Activation of GABA_A_ receptors by pretreatment of PIRC or PRC with a muscimol protected animals from development of AT-evoked seizures (Halonen et al., 1994). The effect of muscimol was unique to PIRC and PRC with no seizure prevention observed when it was placed into areas such as amygdala or hippocampus (Halonen et al., 1994). This suggests that proper functioning of GABA_A_ receptors is critical for prevention of ictal activity in PIRC and PRC, two areas that are hyperexcitable under normal conditions and serving as epilepsy trigger zones (Giacchino et al., 1984). These data demonstrate that ERC is capable of producing focal epileptiform discharges, but also has the necessary functional connections to facilitate propagation of these discharges to other brain areas. Febrile seizures upregulate the expression of GABA_A_ receptors in neonatal granule cells. The increase in GABA_A_receptors causes a reversal in the direction of granule cell migration. Furthermore, preventing GABA-mediated action by using bumetanide, which inhibits that Na,^+^-K^+^-2-Cl^−^ co-transporter, susceptibility to limbic seizures is reduced (Fujiwara-Tsukamoto et al., 2003; Koyama et al., 2012).

ERC function is significantly altered by epileptogenic insults. For example, unilateral damage to ERC caused by amino-oxyacetic acid (AOAA) produced long-lasting (1 month–1.5 year) changes in extracellular responses to white matter stimulation in the superficial layers of the medial ERC. Extracellular responses in slices from AOAA treated animals were prolonged and repetitive and intracellular recordings from residual principal cells revealed prolonged, repetitive excitatory postsynaptic potentials (EPSPs). This pattern differed significantly from the brief EPSPs and single discharges seen in slices from controls. In neurons located in the deep layers of ERC, the initial synaptic response to white matter stimulation did not differ between AOAA and control treated animals. However, in a subset of deep neurons in AOAA treated animals, repetitive action potentials were seen after a brief delay (Scharfman et al., 1998). All together, these changes are consistent with those needed to facilitate the generation of sustained synchronous activity.

## ERC pharmacological studies

As in PIRC/PRC, glutamatergic and GABAergic neurotransmission within ERC are essential for seizure propagation. However, relatively fewer studies have directly manipulated ERC neurotransmitter signaling *in vivo*. In the sections below, we will outline the *in vivo* and *in vitro* pharmacological studies that have established ERC as a key site for seizure origin and propagation.

### Glutamatergic transmission

The role of glutamatergic neurotransmission within ERC is very similar to its role in the PIRC. Not only glutamate antagonists have a differential effect on epileptiform activity depending on the type of receptor blocked, but the effect of the antagonists also differ based on the type of discharges. Glutamatergic transmission has a function in both seizure generation in the deep layers and seizure propagation in the superficial layers of the ERC. Moreover, the process of kindling induces long-lasting changes in glutamatergic neurotransmission within ERC (Cincotta et al., 1991; Lee et al., 1994; Prince et al., 1995; Behr et al., 2001), along with other limbic regions.

### AMPA

In the slice, the role of AMPA-receptor mediated neurotransmission is consistent across ages and methods of inducing epileptiform activity. AMPA antagonists suppress seizure-like events caused by increases in extracellular potassium, bath application of 4-AP, decrease in extracellular magnesium concentration (Borck and Jefferys, 1999; Luhmann et al., 2000) and pilocarpine application (Nagao et al., 1996). Moreover, AMPA antagonists also attenuate a subset of interictal discharges (Avoli et al., 2013). *Ex vivo*, in the isolated guinea pig brain, AMPA receptor blockade completely suppresses seizure activity evoked either electrically from lateral ERC (Federico and MacVicar, 1996) or by 4-AP perfusion (Carriero et al., 2010).

While the effects of *in vivo* manipulation of glutamatergic neurotransmission within ERC on seizure propagation remain understudied, the compelling slice and *ex vivo* data above suggest an instrumental role for AMPA-receptor mediated glutamatergic signaling in seizure generation within ERC.

### NMDA

In contrast to AMPA-receptor mediated transmission, NMDA receptor mediated signaling does not appear to play as consistently vital a role in seizure propagation within ERC. NMDA receptor antagonists may have no effect in slice models (Lücke et al., 1995; Avoli et al., 1996; Nagao et al., 1996; Luhmann et al., 2000), and in the *ex vivo* guinea pig model (Federico and MacVicar, 1996). However, other authors have found that NMDA antagonists decrease the probability and speed of propagation, but do not abolish the propagation completely (Avoli et al., 1994).

Some studies have suggested that NMDA-receptor-mediated transmission plays a particularly important role in the genesis of late epileptiform events, as compared to early events (Zhang et al., 1994). In slices taken from rats that had received status epilepticus, blockade of NMDA receptors partially normalized neuronal activity (Bear et al., 1996; Fountain et al., 1998). As with AMPA-mediated transmission, there is a dearth of data regarding effects of focal entorhinal pharmacological manipulations on seizure propagation. In conclusion, glutamatergic transmission in particular mediated by AMPA receptors is vital for seizure genesis within ERC and propagation both within ERC and to other structures.

### GABA

A failure of the inhibitory control mechanisms that usually dominate interactions between PRC and ERC may underlie seizure initiation and propagation. In the *ex vivo* guinea pig brain, disinhibition by application of bicuculline with concentrations as low as 20 μM resulted in spontaneous seizures that spread to the amygdala more rapidly than electrically evoked seizures (Federico and MacVicar, 1996). Moreover, when compared to electrically-evoked seizures, the distribution area of bicuculline-induced seizures was greater, involving the posterior PIRC and larger areas over the medial amygdala. Both the speed of propagation and the extent of seizures were proportional to the concentration of bicuculline. In this preparation, interictal activity is first detected in ERC, before propagating to other regions and progressing to fast ictal discharges (Uva et al., 2004). Interestingly, during the onset of seizure activity in this model, principal (excitatory) neurons are silent—ictal activity is generated by hypersynchronous and sustained firing of interneurons (Gnatkovsky et al., 2008).

In addition to effects on generation of ictal discharges, blockade of GABA-mediated transmission in the ERC stimulates generation of interictal-like discharges, which consisted of an initial paroxysmal depolarizing shift followed by afterdischarges. The initial component of the interictal discharges, namely, the paroxysmal depolarizing shifts can be blocked in a progressive fashion, while the second component, the afterdischarges, are abolished strictly in all-or-none manner (Jones and Lambert, 1990). Interictal-like discharges represent an intrinsic electrophysiological property of the ERC and support the hypothesis that this region is highly epileptogenic. Similarly, spontaneous sharp waves in the temporal cortex were abolished by administration of GABA antagonists bicuculline (Köhling et al., 1998) or μ-receptor agonists (Louvel et al., 2001), while removal of antagonists restored paroxysmal depolarizing activity (Köhling et al., 1998), effectively suggesting that the synchronization currents were mediated solely by GABA transmission.

The epileptogenecity is associated with changes on the molecular, cellular, and system levels that facilitate generation and propagation of seizures. The implication of these changes can be measured in part as a change in the latency to seizure generation under stimulating conditions. The latency to occurrence of the first seizure-like events after brain slices from SE-subjected animals were placed in Mg^2+^-free artificial cerebrospinal fluid was significantly reduced at 4 and 8 weeks. This provides an evidence for escalation of brain excitability and neuronal hypersynchrony due to adaptations in brain plasticity, predisposing to recurrent epileptic seizures (Holtkamp et al., 2011). Exposure to a convulsant such as 4-AP produces lasting changes in the ERC suggesting plasticity that renders the area epileptogenic. Moreover, different network interactions within the hippocampus-ERC loop characterize control and epileptic slices in the pilocarpine model of epilepsy. For example, ERC-driven ictal discharges in pilocarpine-treated slices occur throughout the experiment and spread to the CA1/subicular area via the temporoammonic path (D'Antuono et al., 2002). These functional changes caused by seizure-induced cell damage lead to the development of epileptic seizures. The process is facilitated by a disinhibition of the ERC due to loss of feed-forward inhibition from the PRC and hippocampal outputs and possibly sustained by the reverberant activity between ERC and CA1 networks that are excited via the temporoammonic path (D'Antuono et al., 2002).

In conclusion, pharmacological means have demonstrated that ERC is an area that is highly susceptible to ictal discharges when exposed to low doses of excitatory compounds.

## Conclusion

In this review, we have described the functional neuroanatomy, pharmacology, and physiology of three limbic-cortical areas: ERC, PRC, and PIRC. These regions are highly interconnected with each other and with other limbic structures, and have features that make them particularly prone to the genesis and propagation of ictal discharges. While a great deal of attention has been paid to subcortical limbic structures in epilepsy (e.g., amygdala, hippocampus), the data reviewed herein clearly underscore the importance of these cortical regions for epileptogenesis.

### Conflict of interest statement

The authors declare that the research was conducted in the absence of any commercial or financial relationships that could be construed as a potential conflict of interest.

## Dedication

We dedicate this article in memory of Dr. Karen Gale, Ph.D. who passed away on August 21, 2014. In addition to her extraordinary contributions to neuroscience, she was most notably the first to discover the region of the brain that triggers epileptic seizures called the Area Tempestas. A remarkable teacher, mentor, and advocate, Dr. Gale helped develop the Interdisciplinary Program in Neuroscience and Georgetown Women in Medicine, both at Georgetown University. Her passion and commitment to the scientific community at all levels will be remembered.

## Abbreviations

4-AP: 4-aminopyridine
AOAA: amino-oxyacetic acid
AMPA: alpha-amino-3-hydroxy-5-methylisoxazole-4-propionic acid
AT: area tempestas
CPP: 3-(2-carboxypiperazin-4-yl)propyl-1-phosphonic acid
EPN: endopiriform nucleus
EPSP: excitatory postsynaptic potentials
ERC: entorhinal cortex
GABA: gamma aminobutyric acid
NBQX: 2,3-dihydroxy-6-nitro-7-sulfamoyl-benzo (f)-quinoxaline
NMDA: N-methyl-D-aspartate
PIRC: piriform cortex
PRC: perirhinal cortex
PSA-NCAM: polysialylated neural cell adhesion molecule
TLE: temporal lobe epilepsy.
